# A logistic regression model to predict the next rabies virus host-shift event

**DOI:** 10.1038/s41598-025-98986-x

**Published:** 2025-06-02

**Authors:** Cassandra Boutelle, Nardus Mollentze, Crystal Gigante, Felipe Rocha, Marco A. N. Vigilato, Daniel G. Streicker, Ryan Wallace

**Affiliations:** 1 Poxvirus and Rabies Branch, United States Centers for Disease Control and Prevention, Atlanta, GA USA; 2 School of Biodiversity, One Health and Veterinary Medicine, University of Glasgow, Glasgow, G12 8QQ UK; 3 MRC-University of Glasgow Centre for Virus Research, Glasgow, G61 1QH UK; 4 Pan American Center for Foot and Mouth Disease and Veterinary Public Health-PANAFTOSA/VPH-PAHO/WHO, Rio de Janeiro, Brazil

**Keywords:** Ecology, Diseases

## Abstract

Rabies virus (RABV) host-shift events (HSEs) are thought to be promoted by viral genomic and ecological factors, but the relative balance of the two is unclear. Using a dataset of 19,170 species pairs that were known or not known to be linked by an HSE, we developed a logistic regression model to explore how biological and ecological characteristics of cross-species infections and their associated RABV variant (RVV) influence HSE risk. The model incorporates relatedness, body temperature, litter size, adult weight, and the broad lineage (bat or canine) of the RABV variant maintained by the reservoir species. Assessed with leave-one-out cross validation, the model identifies known HSEs with 90% accuracy (sensitivity 90%, specificity 82%). The susceptible-reservoir infection on each continent with the highest risk of HSE are coyotes with Canine-associated RVVs (North/Central America, RR = 187.0), culpeos with Canine-associated RVVs (South America, 100.9), dholes with Canine-associated RVVs (Asia, 159.8), arctic foxes with Raccoon Dog RVV (Europe, 115.8), and African wild dogs with Canine-associated RVVs (Africa, 134.8). The results of this model can be used to help predict the next HSE, identify potential cryptic RABV reservoirs, inform contingency actions when a high-risk event is identified, and prepare for importation or incursion events.

**Supplementary Information:**

The online version contains supplementary material available at 10.1038/s41598-025-98986-x.

## Introduction

Rabies virus (RABV) is maintained by numerous reservoir species around the world, most of which are associated with a distinct Rabies Virus Variant (RVV). Although each RVV is mostly maintained by a single reservoir species, cross-species transmissions are frequent^2^. Often, the infected non-reservoir animal will die before further transmitting RABV, however, more rarely transmission is sustained in a new species, resulting in a Host-shift Event (HSE). For example, in North America, Canine-associated RVVs shifted from dogs (*Canis lupus familiaris*) into foxes (arctic - *Vulpes lagopus*, gray - *Urocyon cinereoargenteus*, red - *Vulpes vulpes*) and skunks (spotted - *Spilogale putorius*, striped - *Mephitis mephitis*), while host-shifts from bats resulted in enzootic transmission in skunks and raccoons (*Procyon lotor*)^2^. Furthermore, numerous abortive HSEs have been described in the United States, including bat variants into foxes and skunks, and the Eastern Raccoon RVV into gray foxes^3,4^. HSEs have been observed in other parts of the world as well: *Tadarida brasiliensis* RVV to coatis (*Nasua narica*) in Mexico, Canine-associated RVVs to mongoose (*Herpestes edwardsi*) in India and to ferret-badgers (*Melogale moschata*) in Taiwan^5,6^. These HSEs have changed the way humans interact with wildlife and the precautions we must take around wildlife for both humans and pets. It is important to quickly recognize when HSEs occur, or have the potential to occur, so that proper public health and wildlife management measures can be taken.

HSEs are promoted through two mechanisms: viral genomic changes that increase the likelihood of onward transmission after cross-species transmission or ecological changes that create or increase the frequency of inter- and/or intra-specific interactions^7^. While the RABV genome is conserved and has a relatively low mutation rate for an RNA virus, molecular and antigenic changes are often detected through robust surveillance programs and can be used to characterize the enzootic transmission cycle. RABV mutations have been purported to predispose the virus to host-shifts in certain species (e.g. *Eptesicus fuscus* RVVs may be more molecularly-primed for sustained transmission in coyotes [*Canis latrans*] and striped skunks)^3^. Similarly, establishing circulation in one host species may lead to evolutionary changes that pre-adapt RABV to other host species^8^. Alternatively, wildlife (and occasionally domestic animals) may incur ecological changes that favor an HSE in the absence of any phenotypically relevant viral genome changes, such as increases in population density or incursions/translocations into new ecosystems.

Potential cryptic reservoir species of RABV have been identified using machine learning algorithms trained on the ecological and life history characteristics of known RABV reservoirs^9^. However, these algorithms considered neither virus variant effects on the likelihood of host-shifts nor pairwise physiological differences between candidate reservoir and recipient species. A recent meta-analysis of experimental cross-species RABV infections characterized the biological and ecological associations of extant RVVs which may predispose certain RABV transmission events to be more host-shift-favorable^10^. Biological characteristics like body temperature, body temperature difference between reservoir and susceptible species, and phylogenetic relatedness between species were associated with changes to the outcomes of infections that were hypothesized to alter the likelihood of forward transmission of RABV infections^10^. These new findings provide insight into the ecological host-shift mechanism and are worthy of further exploration.

We developed a multivariable logistic regression model to explore the ecological host-shift mechanism and to predict the probability that a susceptible-reservoir RABV infection (a hypothetical infection pair, consisting of a susceptible species and a RABV reservoir species) will result in an HSE based on host-variant relationships of biological and ecological characteristics. The results of this model are intended to inform three primary actions: (1) Inform contingency actions when high risk host-variant combinations are identified, (2) Identify high-risk species potentially involved in cryptic enzootic rabies virus transmission cycles, and (3) Identify high-risk host-variant combinations that may evolve through events such as global climate change or the translocation of animals.

## Methods

We sought to develop a model which distinguished pairs of mammalian species which had or had not been linked by historical RABV HSEs. We defined susceptible species (i.e., candidate recipients of host-shifts from reservoirs) as any terrestrial mammalian species. Those species which are currently known to sustain a RABV transmission cycle are considered susceptible to other RVVs. Established RVVs were identified by conducting a search on PubMed for articles published before 1 January 2024 using keywords “rabies” and “variant” for known current and historic reservoir species and identifying the lineage of RVVs through epidemiologic or phylogenetic evidence. Only variants with phylogenetic evidence of a novel RVV or well-documented epidemiologic evidence indicating significant transmission among a new species (i.e., more than common spillover from a known reservoir) were included. Table S1 and Table S2 describe the RVVs and respective reservoir species included in the model, their lineage, status, location, and supporting evidence.

In efforts to include all known HSEs, the model includes both current and historic RVVs. The status of these RVVs is important to account for when drawing inferences from the model. Active status indicates current maintenance by the reservoir species. Natural extinction indicates that RABV was once maintained by the reservoir species, but that RVV naturally died out without human intervention. Anthropogenic extinction indicates that RABV was once maintained by the reservoir species, but that RVV was eliminated by humans through vaccination or other means. Abortive indicates that there is epidemiologic or phylogenetic evidence of a new RVV in the reservoir species, but the RVV was not maintained by the species for a prolonged period of time. Intermittent indicates that there is epidemiologic or phylogenetic evidence of a new RVV in the reservoir species, and the RVV is not consistently observed, but re-emerges in cycles. Extinct and abortive variants were included in the model if there was sufficient evidence, since these RVVs resulted from HSEs. Although Oceania is historically rabies-free, the model was run with susceptible species in Oceania infected with Canine-associated RVVs to explore the risk of incursion and importation^11^.

19,170 susceptible-reservoir infections were used to fit the model, including information from 912 susceptible species and 67 RVVs. All analyses and visualizations were created using R version 4.3.1 or QGIS version 3.40.4^12,13^. Susceptible species were selected based on those species documented in the AnAge database^14^. Data for all examined characteristics were gathered from AnAge, except relatedness data which were gathered from the TimeTree database^15^. These databases were selected for use in the model for their robust number of mammalian species and the quality and variety of characteristics collected as well as their public availability. If a characteristic was missing for a specific species, the value was imputed using the next closest relative(s) with information available. Imputed values were calculated by taking the average value of the species’ relatives at the lowest classification level (genus, family, or order) where data for that characteristic were available.

**Fig. 1:**
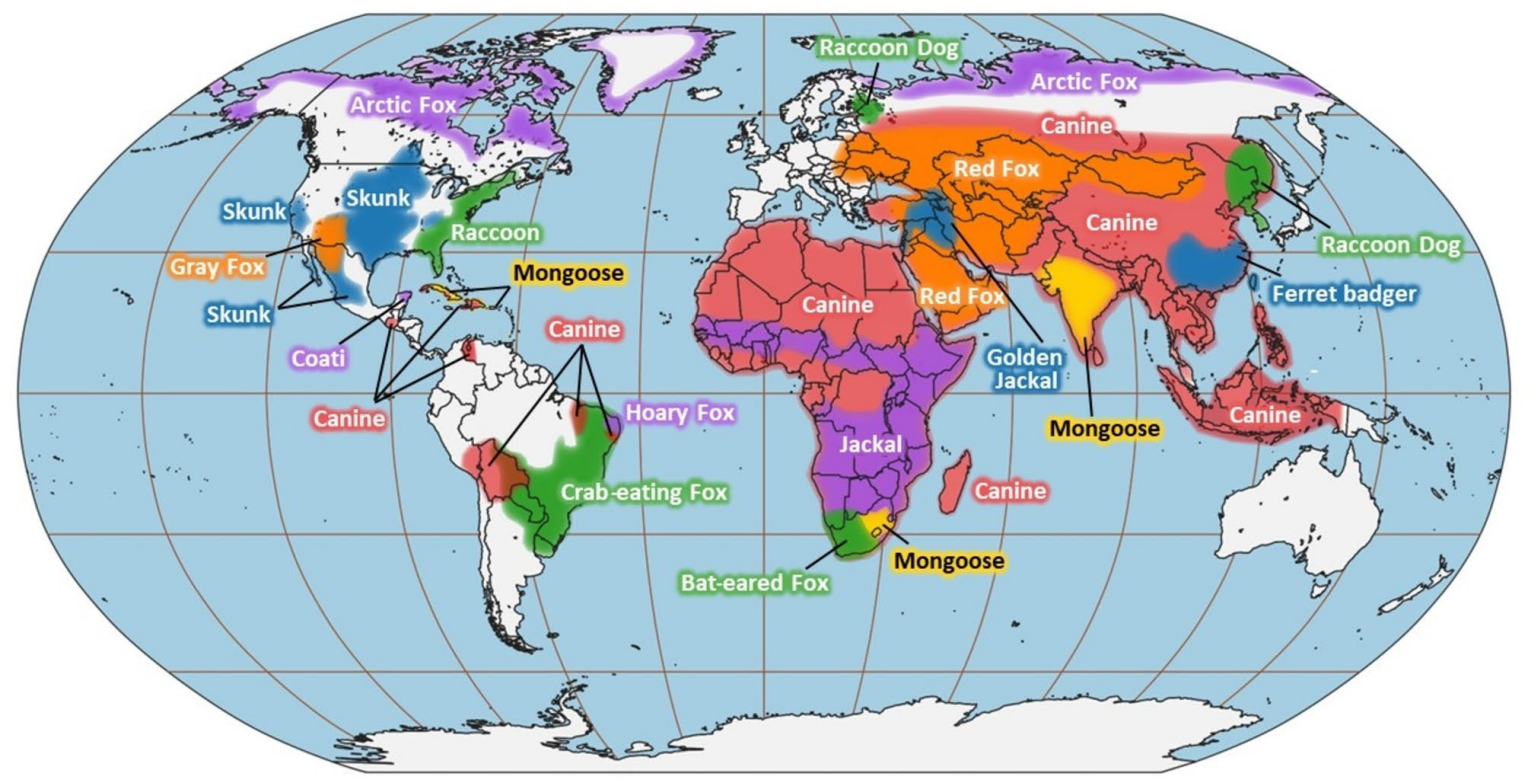
Approximate geographic area of RVV transmission areas for known terrestrial RABV reservoir species. The true extent of RVV transmission areas is limited to the level of surveillance conducted and shared by global rabies programs. For reservoir species where the extent of RABV spread is not well described, the transmission areas were extrapolated using habitat range information^1^. Map made using Natural Earth and QGIS version 3.40.4.

It was assumed that in each susceptible-reservoir infection, the RVV is transmitted by its associated reservoir species (e.g. Eastern Raccoon RVV is assumed to be transmitted by a raccoon). Susceptible-reservoir infections included in the model consist of pairs where the susceptible species inhabits the same continent where the RVV in question circulates (or circulated historically, in the case of extinct variants). For example, the pairing coyote and Eurasian Gray Wolf RVV was not used to fit the model because although both species inhabit North America, gray wolves (*Canis lupus*) were only a RABV reservoir on the Eurasian continent historically, so coyote-Eurasian Gray Wolf RVV would not be a valid susceptible-reservoir infection. Habitat range for susceptible species was gathered from the ASM Mammal Diversity database which was selected for its robust species list and ease of extraction of location information^16^. Figure 1 depicts the approximate geographic area of all known active terrestrial RABV reservoir species based on information in the supporting evidence for RVVs as described in Table S1 and the reservoir species habitat range according to IUCN^1^.

Predictor variables, described in Table 1, were chosen from biological and ecological characteristics that were found to be associated with longer incubation or viral shedding periods and/or increased virus excretion during experimental cross-species infections^10^. For the purposes of this analysis, we assume these changes in infection outcomes are likely to increase the likelihood of forward transmission of RABV in the recipient host species. Covariates included: susceptible species’ average body temperature (degrees Kelvin), higher body temperature of susceptible species relative to the reservoir species (average body temperature difference in degrees Kelvin), and increased relatedness between susceptible and reservoir species (phylogenetic patristic distance)^10^.

**Table 1:** Model parameters and definitions selected for their impact on the forward transmission of RABV. Relatedness was retrieved from timetree^15^. Temperature, litter size, and weight were retrieved from AnAge^14^. Temperature difference and weight difference was calculated from values retrieved from AnAge. Weight difference categories were calculated using the quantile function from the stats R package^12^. Lineage was determined by the supporting evidence for the RVV.

Model parameter		Definition
Relatedness		Phylogenetic relatedness of the susceptible and reservoir species (phylogenetic patristic distance)
Temperature		Average body temperature of the susceptible species (degrees Kelvin)
Temperature difference		Difference in average body temperature between the reservoir and susceptible species (degrees Kelvin)
Litter size		Average litter size of the susceptible species (individuals)
Weight		Average adult weight of the susceptible species log transformed to conform to the normality assumption (g)
Weight difference	Low	Difference in average adult weight (g) between the reservoir and susceptible species in the lower 25% (reservoir species is lighter than susceptible species)
	High	Difference in average adult weight (g) between the reservoir and susceptible species in the upper 25% (reservoir species is heavier than susceptible species)
	Reference	Difference in average adult weight (g) between the reservoir and susceptible species in the middle 50% (reservoir species is similar weight as susceptible species)
Lineage		Lineage of the RVV transmitted by the reservoir species (1 = Bat, 0 = Canine)

Litter size (individuals, log transformed) was selected as a proxy for intra-species interaction, since population density data was limited. The more often an animal interacts with other animals of its species, the more likely it would be that they infect another animal if they were rabid^17,18^. Average adult body weight (g, log transformed) and difference in average adult weight (g) were included to consider the likelihood of forward transmission for those animals that are very large or very small. We hypothesized that if an animal is very small, like mice and squirrels, it would be less likely to survive an attack by a rabid animal long enough to develop infection. Conversely, for very large animals, like elephants, it is less likely that a bite from a rabid animal will be deep enough to manifest in infection. For those animals that are more similar in size, we assume that an animal would be more likely to survive an attack by rabid animal long enough to develop infection and potentially transmit RABV forward. Average adult weight difference was categorized based on quantiles which were calculated using the quantile function of the stats R package^12^. Low indicated the reservoir species is lighter than the susceptible species (in the lowest 25% of model weight differences), high indicated the reservoir species is heavier than the susceptible species (in the highest 25% of model weight differences), and the reference group were reservoir and susceptible species that are similar in weight (between 25% and 75% of model weight differences). A binary variable for lineage of the RVV maintained by the reservoir species was included to account for any differences between bat and canine derived RVVs^7^.

Additional variables that may reflect host ecological characteristics were also considered as model parameters: population density, average longevity, age of maturity, social behavior, diet, and presence of incisor teeth^14,16^. Some of these characteristics were excluded purely due to limited data availability. Backwards elimination was used to assess each variable’s impact on the model. A variable was selected to remain in the model if the change on model outcome was more than 10%, the analysis of variance was significant to 0.05 and VIFs were not significantly high (less than 10), or the variable was deemed critical to include in the model based on expert opinion. The car R package was used to conduct analysis of variance using the Wald test statistic and to calculate VIFs^19^.

A multivariable logistic regression model was selected since the primary outcome of interest was a binary variable indicating whether each susceptible-reservoir infection had ever been involved in a documented HSE and was run using the glm function of the stats R package^12^. Known HSEs included in the model are shown in Table 2. These were identified through the evidence supporting the list of known RVVs.

**Table 2:** Known HSEs based on epidemiologic and phylogenetic evidence.

Known Host Shift Events		
Susceptible species	Rabies Virus Variant	Event(s)
Red fox (*Vulpes vulpes*)	Arctic fox	Ontario, Canada^38^
Gray Fox (*Urocyon cinereoargenteus*)	California skunk	California, USA^36^
Arctic Fox (*Vulpes lagopus*)	Canine-associated	Arctic^50^
Bat-eared fox (*Otocyon megalotis*)	Canine-associated	South Africa^42^
Black-backed jackal (*Canis mesomelas*)	Canine-associated	Southern Africa^43,44^
Coyote (*Canis latrans*)	Canine-associated	North America^28^
Crab-eating fox (*Cerdocyon thous*)	Canine-associated	Brazil^40^
Ferret-badger (*Melogale moschata*)	Canine-associated	Taiwan, China^6,51^
Golden jackal (*Canis aureus*)	Canine-associated	Europe/Asia^46^
Gray Fox (*Urocyon cinereoargenteus*)	Canine-associated	North America^28^
Gray wolf (*Canis lupus*)	Canine-associated	Europe/Asia^49^
Hoary fox (*Lycalopex vetulus*)	Canine-associated	Brazil^52^
Mongoose (*Urva auropunctata*,* Herpestes edwardsi*,* Cynictis penicillate*)	Canine-associated	Puerto Rico, USA; India; Africa^41,45,50^
Raccoon dog (*Nyctereutes procyonoides*)	Canine-associated	Europe, Asia^47^
Red fox (*Vulpes vulpes*)	Canine-associated	USA; Turkey; Europe/Asia^26,47,48^
Side-striped jackal (*Canis adustus*)	Canine-associated	Southern Africa^43^
Spotted skunk (*Spilogale putorius*)	Canine-associated	Mexico^27^
Striped skunk (*Mephitis mephitis*)	Canine-associated	USA^27^
Gray Fox (*Urocyon cinereoargenteus*)	Eastern raccoon	Maine, USA^4^
Striped skunk (*Mephitis mephitis*)	*Eptesicus fuscus* W1	Arizona, USA^3^
Gray Fox (*Urocyon cinereoargenteus*)	*Eptesicus fuscus* W2	Arizona, USA^3^
Raccoon (*Procyon lotor*)	*Lasiurus cinereus*	USA^50^
Spotted skunk (*Spilogale putorius*)	*Lasiurus cinereus*	Mexico^27^
Striped skunk (*Mephitis mephitis*)	*Lasiurus cinereus*	North America^53^
Gray Fox (*Urocyon cinereoargenteus*)	*Myotis lucifugus*	Oregon, USA^37^
Coati (*Nasua narica*)	*Tadarida brasiliensis*	Yucatan, Mexico^39^
Dog (*Canis lupus familiaris*)	Texas/Mexico Coyote	Texas, USA^54^

The model was used to approximate risk of a susceptible-reservoir infection leading to an HSE, since the occurrence of HSE is rare in the data. To determine the optimal threshold for considering a susceptible-reservoir infection high risk, a sensitivity analysis was conducted with risk cut-offs at intervals of 0.001. The high-risk threshold was selected based on the risk cut-off with the highest average of sensitivity and specificity. For ease of communication, this threshold was used as the reference value to calculate a risk ratio for each susceptible-reservoir infection. Every susceptible-reservoir infection with a risk ratio greater than one was considered at high-risk for an HSE. Model accuracy was assessed using leave-one-out cross validation^20^.

Average risk for each susceptible species was calculated by taking the average of all susceptible-reservoir infections involving that susceptible species. The same was done for each RVV. These average risks were calculated to assess which susceptible species may be priority candidates to target when increasing wildlife surveillance, or which reservoir species should be monitored for habitat changes and translocation events.

Model results were applied to a geographic analysis to determine what areas of the world are at the highest risk for experiencing an HSE. The cumulative risk for host-shift was calculated by summing the risk of all high-risk susceptible-reservoir infections in a geographic area using the fasterize R package^21^. The geographic range of each susceptible-reservoir infection was determined by the habitat range of the susceptible species^1^. For those susceptible species that inhabit multiple continents, risk was only added to their habitat range where the susceptible-reservoir infection may occur. For example, although red foxes inhabit parts of Africa, Asia, Europe, and North America, their risk of host-shift from Arizona/Mexico Gray Fox RVV is only accounted for in their North American habitat range since the RVV is not maintained on other continents.

## Results

The final variables selected for the model were (a) relatedness of the susceptible and reservoir species, (b) average body temperature of the susceptible species, (c) difference in average body temperature between the reservoir and susceptible species, (d) litter size of the susceptible species, (e) average adult weight of the susceptible species, (f) difference in average adult weight between the reservoir and susceptible species, and (g) the lineage of the RVV transmitted by the reservoir species. These variables significantly impacted the model fit and were deemed critical to consider when evaluating the risk of an HSE (Table S3). Several other characteristics were considered but ultimately excluded from the model due to lack of data availability or insignificant impact on the overall model determined through backwards elimination.

**Fig. 2:**
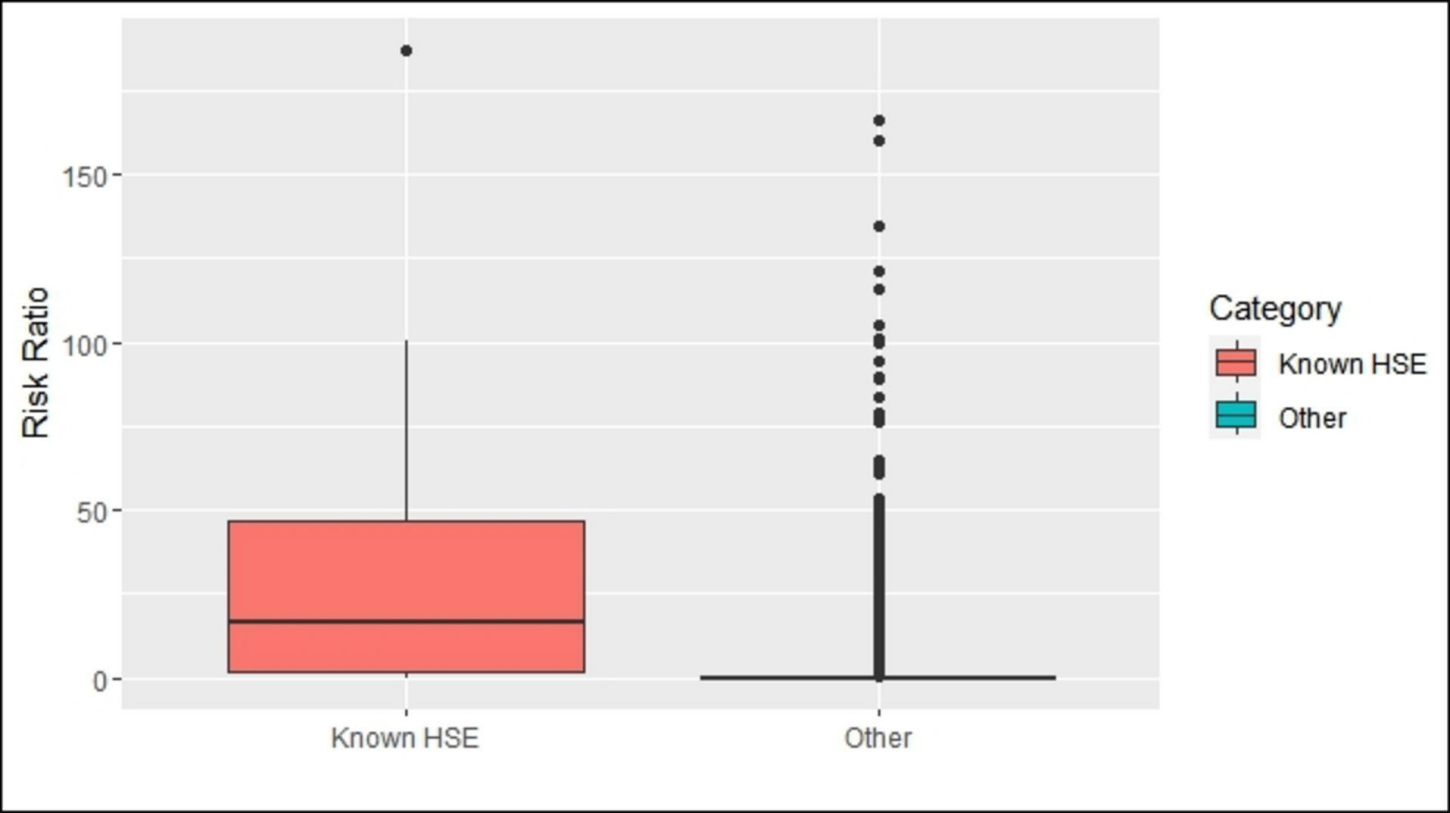
Known HSEs have an average risk ratio of 34.5, while other susceptible-reservoir infections have an average risk ratio of 0.7. Most other susceptible-reservoir infections have a risk ratio less than 1, with a handful of outliers.

The sensitivity analysis showed the highest average sensitivity and specificity using a risk cut-off of 0.002. At this threshold, the model identified known HSEs with 90% accuracy (sensitivity 90%, specificity 82%). R-squared value was 0.33 and leave-one-out cross validation resulted in an average accuracy of 90%. The average risk ratio for known HSEs was 34.5, while the average risk ratio for other susceptible-reservoir infections was 0.7 (Fig. 2). Full model inputs and results can be found in Dataset S1.

Table 3 shows the 20 susceptible-reservoir infections at greatest risk of an HSE for each continent. Table 4 lists the susceptible species with highest average risk of an HSE on each continent, and Table 5 lists the average risk of an HSE from the RVVs on each continent. These tables only include RVVs that are active on the continent and exclude any susceptible-reservoir infections where it is suspected that the susceptible species also plays a role in the maintained transmission of the RVV.

**Table 3:** Susceptible-reservoir infections with the highest risk of a host-shift event in each continent. Only RVVs that are active on the continent are included on these lists. Known HSE indicates that the susceptible-reservoir infection has a documented host-shift event. Scientific names for susceptible species can be found in dataset S1. Various species of skunk are suspected to play a role in the maintained transmission of skunk-associated RVVs in North America, so susceptible-reservoir infections where both species are skunks are excluded from results. *Species actively maintains an RVV (not necessarily the RVV in the listed susceptible-reservoir infection) on the continent.

Susceptible species	Variant	RR	Known HSE
**North and Central America**			
Coyote	Canine-associated	187.0	✓
Arctic Fox*	Canine-associated	100.6	✓
Gray wolf	Canine-associated	70.4	
Bush dog	Canine-associated	63.1	
Striped skunk*	Eastern Raccoon	62.1	
Coati*	Eastern Raccoon	47.7	
Coyote	Arctic Fox	42.7	
Coyote	Arizona/Mexico Gray Fox	42.2	
Red fox	Canine-associated	38.3	✓
Kit fox	Arctic Fox	37.6	
Gray Fox*	Canine-associated	36.2	✓
Swift fox	Arizona/Mexico Gray Fox	26.4	
Swift fox	Canine-associated	26.4	
Crab-eating raccoon	Eastern Raccoon	25.0	
Kit fox	Arizona/Mexico Gray Fox	24.1	
Kit fox	Canine-associated	24.1	
Hooded skunk*	Eastern Raccoon	23.3	
Coyote	Eastern Raccoon	22.1	
Hooded skunk*	Yucatan Coati	21.5	
Red fox	Eastern Raccoon	20.0	
**South America**			
Culpeo	Canine-associated	100.9	
Maned wolf	Canine-associated	88.8	
Pampas fox	Canine-associated	78.3	
Argentinian gray fox	Crab-eating Fox	64.6	
Bush dog	Canine-associated	63.1	
Argentinian gray fox	Brazil Hoary Fox	60.7	
Argentinian gray fox	Canine-associated	52.5	
Gray Fox	Crab-eating Fox	38.7	
Crab-eating fox*	Canine-associated	38.5	✓
Hoary fox*	Canine-associated	36.5	✓
Gray Fox	Canine-associated	36.2	
Culpeo	Crab-eating Fox	35.5	
Culpeo	Brazil Hoary Fox	33.2	
Pampas fox	Crab-eating Fox	26.6	
Pampas fox	Brazil Hoary Fox	24.9	
Bush dog	Crab-eating Fox	17.9	
Bush dog	Brazil Hoary Fox	16.7	
South american coati	Crab-eating Fox	13.0	
South american coati	Canine-associated	12.1	
Hoary fox	Crab-eating Fox	11.6	
**Africa**			
African wild dog	Canine-associated	134.8	
Side-striped jackal*	Canine-associated	97.9	✓
Black-backed jackal*	Canine-associated	79.6	✓
Red fox	Black-backed Jackal	42.0	
Red fox	Side-striped Jackal	42.0	
Bat-eared fox*	Black-backed Jackal	41.1	
Bat-eared fox*	Side-striped Jackal	41.1	
Red fox	Canine-associated	38.3	
Bat-eared fox*	Canine-associated	37.5	✓
Cape fox	Black-backed Jackal	33.6	
Cape fox	Cape fox	33.6	
Side-striped jackal*	Black-backed Jackal	30.8	
Cape fox	Canine-associated	30.6	
Black-backed jackal*	Side-striped Jackal	24.3	
Side-striped jackal*	Bat-eared Fox	19.8	
African clawless otter	Canine-associated	16.6	
Black-backed jackal*	Bat-eared Fox	15.5	
Tenrec	Black-backed Jackal	10.2	
Tenrec	Side-striped Jackal	10.2	
Red fox	Bat-eared Fox	10.0	
**Asia**			
Dhole	Canine-associated	159.8	
Arctic Fox*	Raccoon Dog	115.8	
Arctic Fox*	Canine-associated	100.6	✓
Arctic Fox*	Golden Jackal	99.5	
Raccoon dog*	Canine-associated	95.1	✓
Raccoon dog*	Golden Jackal	94.0	
Golden jackal*	Canine-associated	75.3	✓
Gray wolf	Canine-associated	70.4	✓
Dhole	Golden Jackal	49.8	
Corsac fox	Eurasian Red Fox	45.5	
Red fox*	Raccoon Dog	45.2	
Corsac fox	Arctic Fox	44.7	
Arctic Fox*	Eurasian Red Fox	40.8	
Dhole	Raccoon Dog	38.9	
Red fox*	Canine-associated	38.3	✓
Red fox*	Golden Jackal	37.8	
Corsac fox	Raccoon Dog	36.4	
Corsac fox	Canine-associated	30.8	
Corsac fox	Golden Jackal	30.4	
Raccoon dog*	Arctic Fox	29.1	
**Europe**			
Arctic Fox*	Raccoon Dog	115.8	
Arctic Fox*	Golden Jackal	99.5	
Corsac fox	Eurasian Red Fox	45.5	
Red fox*	Raccoon Dog	45.2	
Corsac fox	Arctic Fox	44.7	
Arctic Fox*	Eurasian Red Fox	40.8	
Red fox*	Golden Jackal	37.8	
Corsac fox	Raccoon Dog	36.4	
Corsac fox	Golden Jackal	30.4	
Golden jackal*	Raccoon Dog	14.7	
Red fox*	Arctic Fox	14.4	
Golden jackal*	Arctic Fox	13.5	
Golden jackal*	Eurasian Red Fox	13.3	
European polecat	Raccoon Dog	7.1	
European polecat	Arctic Fox	6.6	
European polecat	Eurasian Red Fox	6.5	
European polecat	Golden Jackal	6.4	
Marbled polecat	Raccoon Dog	5.6	
Wild cat	Golden Jackal	5.5	
Marbled polecat	Arctic Fox	5.1	

**Table 4:** The ten highest average risk of HSE for susceptible species by continent. Reservoir indicates that that species is (or historically has been) a reservoir on that continent. Scientific names for susceptible species can be found in Dataset S1.

Susceptible species	Average RR	Reservoir
**North and Central America**		
Arctic Fox	15.8	✓
Coyote	12.9	✓
Hooded skunk	9.6	✓
Swift fox	8.8	
Striped skunk	8.7	✓
Kit fox	8.1	
Bush dog	7.7	
Hog-nosed skunk	6.4	✓
Red fox	5.6	✓
Gray Fox	4.9	✓
**South America**		
Argentinian gray fox	13.9	
Culpeo	12.2	
Pampas fox	11.2	
Bush dog	8.5	
Gray Fox	7.1	
Maned wolf	6.5	
Crab-eating fox	4.8	✓
Hoary fox	4.6	✓
Nutria	4.1	
South American coati	3.5	
**Africa**		
Side-striped jackal	49.5	✓
Bat-eared fox	39.9	✓
Black-backed jackal	39.8	✓
African wild dog	36.8	
Red fox	26.7	
Cape fox	26.4	
Tenrec	9.8	
European polecat	6.8	
Ruppell’s sand fox	6.7	
Sand cat	5.2	
**Asia**		
Arctic Fox	64.2	✓
Raccoon dog	54.5	✓
Dhole	52.2	
Corsac fox	33.5	
Golden jackal	29.9	✓
Red fox	25.7	✓
Smooth-coated otter	17.9	
Dog	11.4	✓
Gray wolf	9.6	✓
Ruppell’s sand fox	6.3	
**Europe**		
Arctic Fox	83.8	✓
Corsac fox	38.0	
Red fox	35.2	✓
Golden jackal	34.9	✓
Dog	15.2	✓
Gray wolf	12.8	✓
European polecat	6.6	
Old World badger	6.3	
Wolverine	5.2	
Marbled polecat	5.1	
**Oceania**		
Cat	4.4	
Rat	0.8	
Southern elephant seal	0.3	
Guinea pig	0.2	
Swine	0.2	
Antarctic fur seal	0.1	
Cattle	0.1	
Crabeater seal	0.1	
Leopard seal	0.1	
Ross seal	0.1	

**Table 5:** Average risk of HSE caused by the RVVs of each continent. Known HSE indicates that the RVV caused a documented HSE on that continent. †Variant is extinct or abortive on the continent.

Variant	Average RR	Known HSE
**North and Central America**		
Canine-associated	3.0	✓
Texas/Mexico Coyote†	2.3	✓
Eastern Raccoon	2.1	✓
Oregon Gray fox†	1.9	
South Central Skunk	1.6	
Flagstaff Skunk	1.6	
Yucatan Coati	1.2	
Arctic Fox	1.1	✓
North American Red Fox†	1.1	
Ontario Red Fox†	1.1	
Texas/Mexico Gray Fox†	1.0	
Arizona/Mexico Gray Fox	1.0	
California Gray Fox†	1.0	
Maine Gray Fox†	1.0	
Flagstaff Gray Fox†	1.0	
California Skunk	0.8	✓
North Central Skunk	0.8	
Central Mexico Skunk	0.8	
*Parastrellus hesperus*	0.4	
*Lasiurus noctivagans*	0.4	
Baja California Skunk	0.4	
Sonora Skunk	0.4	
*Myotis lucifugus*	0.3	✓
*Myotis californicus*	0.3	
*Myotis evotis*	0.3	
*Myotis keenii*	0.3	
*Myotis leibii*	0.3	
*Myotis septentrionalis*	0.3	
*Lasiurus xanthinus*	0.3	
*Lasiurus intermedius*	0.3	
*Myotis yumanensis*	0.3	
*Myotis thysanodes*	0.3	
*Myotis volans*	0.3	
*Mytois ciliolabrum*	0.3	
*Antrozous pallidus*	0.3	
*Nycticeius humeralis*	0.3	
*Lasiurus cinereus*	0.2	✓
Grenada Mongoose	0.2	
*Desmodus rotundus*	0.2	
*Macrophyllum macrophyllum*	0.2	
*Lasiurus seminolus*	0.2	
*Pipistrellus subflavus*	0.2	
*Corynorhinus townsendii*	0.2	
*Lasiurus borealis*	0.2	
Cuba Mongoose	0.2	
*Eptesicus fuscus*	0.2	✓
*Tadarida brasiliensis*	0.2	✓
*Eptesicus furinalis*	0.2	
*Molossus molossus*	0.2	
*Nyctinomops ssp.*	0.2	
*Artibeus lituratus*	0.2	
Puerto Rico Mongoose	0.1	
**South America**		
Canine-associated	3.2	✓
Crab-eating fox	1.4	
Brazil Hoary fox	1.1	
*Histiotus sp.*	0.4	
*Lasiurus cinereus*	0.2	
*Desmodus rotundus*	0.2	✓
*Pipistrellus subflavus*	0.2	
*Eptesicus fuscus*	0.2	✓
*Tadarida brasiliensis*	0.2	✓
*Eptesicus brasiliensis*	0.2	
*Eptesicus furinalis*	0.2	
*Molossus molossus*	0.2	
*Nyctinomops ssp.*	0.2	
*Artibeus lituratus*	0.1	
**Africa**		
Canine-associated	1.9	✓
Black-backed Jackal	0.9	
Side-striped Jackal	0.8	
Bat-eared Fox	0.4	
Africa Mongoose	0.2	
**Asia**		
Eurasian Gray Wolf†	2.7	
Canine-associated	2.5	✓
Golden Jackal	1.5	
Raccoon Dog	1.2	
Arctic Fox	0.7	
Eurasian Red Fox	0.7	
Turkey Red Fox†	0.7	
Taiwan Ferret-badger	0.3	
India Mongoose	0.2	
**Europe**		
Eurasian Gray Wolf†	3.6	
Canine-associated variants†	3.3	✓
Raccoon Dog	2.2	
Golden Jackal	1.9	
Eurasian Red Fox	1.2	
Turkey Red Fox†	1.2	
Arctic Fox	1.0	
**Oceania**		
Canine-associated (hypothetical)	0.2	

**Fig. 3:**
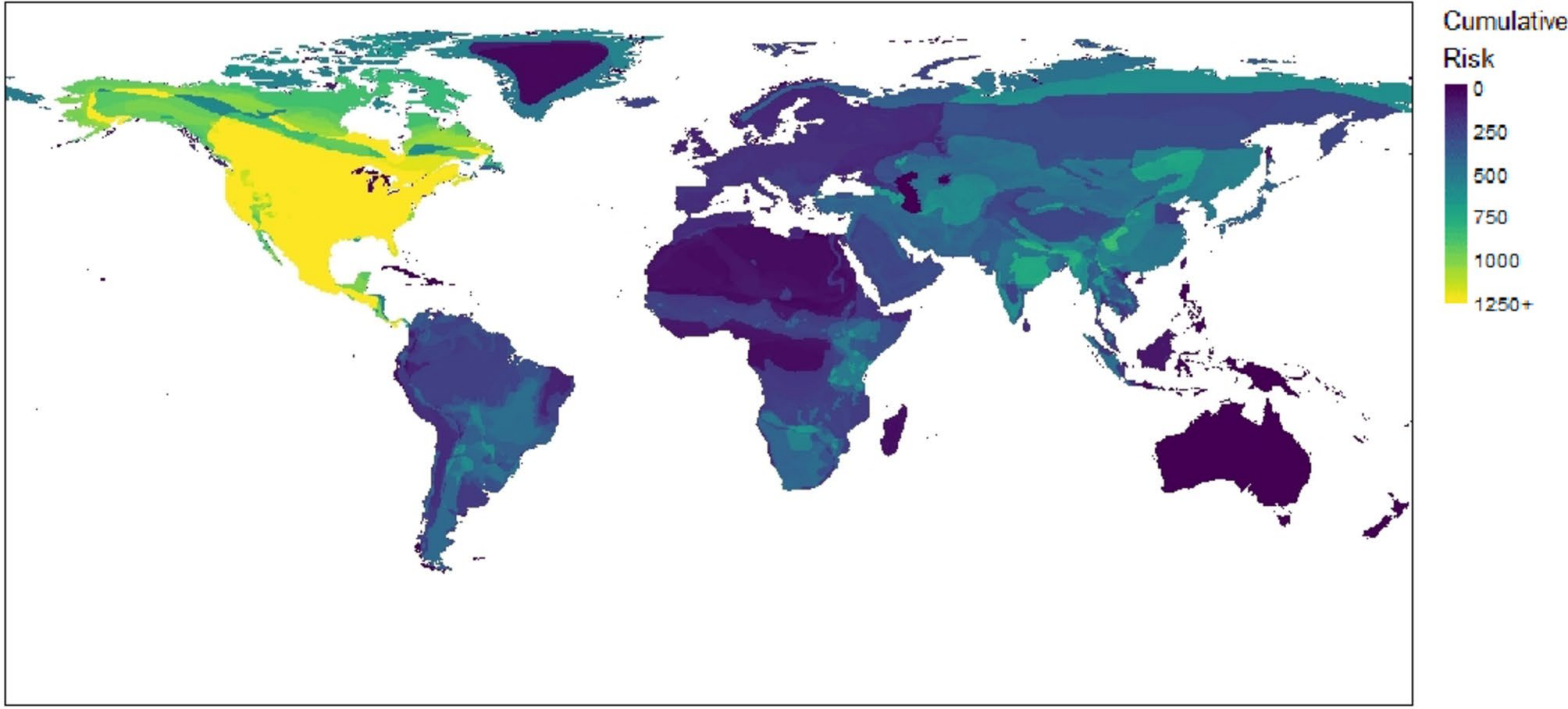
Cumulative risk of HSE for high-risk susceptible-reservoir infections displayed by geographic range of the susceptible species. Extinct and abortive variants are excluded. Susceptible-reservoir infections with RR > 1 are considered high-risk. The cumulative risk of an area is not only affected by the magnitude of risk the susceptible species in that area have, but also the number of susceptible species and potential susceptible-reservoir infections that were considered in the model. The highest cumulative risk is seen in North America. Twenty terrestrial and seventeen bat RVVs were identified in North America (compared to the continent with the next highest number of RVVs, South America with nine) which partially explains why North America has such a high cumulative risk. Other areas with high cumulative risk include central South America, Sub-Saharan Africa, and much of Asia. In general, and partially as a result of the model design, areas with higher biodiversity tend to have higher cumulative risk of HSEs, since there are a greater number of susceptible species with the opportunity for infection. Map made using Esri World Continents from ArcGIS Hub and IUCN spatial data^1^.

Results of the geographic cumulative risk analysis are shown in Fig. 3. The map indicates that North America is at highest risk for an HSE to occur. Sections of central South America, Sub-Saharan Africa, and Asia are also at relatively high risk. Areas where no high-risk susceptible-reservoir infections (RR > 1) occur are shown as having zero risk on the map.

### North & Central America and the Caribbean

In North and Central America, Canine-associated RVVs are associated with the six susceptible-reservoir infections at highest risk for an HSE. The susceptible species at highest risk of an HSE from Canine-associated RVVs are coyotes (RR = 187.0), arctic foxes (*Vulpes lagopus*, 100.6), gray wolves (70.4), bush dogs (*Speothos venaticus*, 63.1), red foxes (38.3), and gray foxes (36.2). Of these species, coyotes, arctic foxes, red foxes, and gray foxes have previously documented HSEs from Canine-associated RVVs, and arctic foxes and gray foxes are active reservoir species of a canine RABV-derived lineage in North America.

The susceptible species with the highest average risk from all North and Central American and Caribbean RVVs are arctic foxes (average RR = 15.8), coyotes (12.9), hooded skunks (*Mephitis macroura*, 9.6), swift foxes (*Vulpes velox*, 8.8), and striped skunks (*Mephitis mephitis*, 8.7). Arctic foxes, hooded skunks, and striped skunks are known active reservoir species, and coyotes are historic reservoir species. The RVVs with the highest average risk are Canine-associated RVVs (3.0), Texas/Mexico Coyote (historic; 2.3), Eastern Raccoon (2.1), Oregon Gray Fox (abortive; 1.9), and South Central Skunk (1.6). Of these reservoir species, only gray foxes are not responsible for a documented HSE.

### South America

Canine-associated RVVs are also responsible for the highest risk interactions in South America. The susceptible species at highest risk are culpeos (*Lycalopex culpaeus*, RR = 100.9), maned wolves (*Chrysocyon brachyurus*, 88.8), pampas foxes (*Lycalopex gymnocercus*, 78.3), bush dogs (63.1), Argentinian gray foxes (*Lycalopex griseus*, 52.5), crab-eating foxes (*Cerdocyon thous*, 38.5), hoary foxes (*Lycalopex vetulus*, 36.5), and gray foxes (36.2). Crab-eating foxes and hoary foxes are active reservoir species in South America with documented HSEs from Canine-associated RVVs.

The susceptible species with the highest average risk are Argentinian gray foxes (average RR = 13.9), culpeos (12.2), pampas foxes (11.2), bush dogs (8.5), and gray foxes (7.1). None of these species are known reservoirs in South America. The RVVs with average risk greater than one are Canine-associated RVVs (average RR = 3.2), Brazil Crab-eating Fox (1.4), and Brazil Hoary Fox (1.1).

### Africa

In Africa, the highest risk interactions are African wild dogs (*Lycaon pictus*) from Canine-associated RVVs (RR = 134.8), side-striped jackals (*Canis adustus*) from Canine-associated RVVs (97.9), black-backed jackals (*Canis mesomelas*) from Canine-associated RVVs (79.6), red foxes from Black-backed Jackal RVV (42.0), and red foxes from Side-striped Jackal RVV (42.0). Black-backed jackals and side-striped jackals are known reservoir species that have a documented HSE from Canine-associated RVVs.

The susceptible species with the highest average risk are side-striped jackals (average RR = 49.5), bat-eared foxes (*Otocyon megalotis*, 39.9), black-backed jackals (39.8), African wild dogs (36.8), and red foxes (26.7). For known RVVs, Canine-associated RVVs have the highest risk for HSE (average RR = 1.9).

### Asia

The highest risk interaction in Asia is a dhole (*Cuon alpinus*) infected with Canine-associated RVVs (RR = 159.8). Arctic foxes infected with Canine-associated RVVs (100.6) and raccoon dogs (*Nyctereutes procyonoides*) infected with Canine-associated RVVs (95.1) are both documented host-shifts which are predicted to have high risk by the model. Arctic foxes and raccoon dogs infected with Golden Jackal RVV are also at high risk (99.5 and 94.0). Arctic foxes and raccoon dogs are known reservoirs in Asia, both with documented HSEs from Canine-associated RVVs.

The susceptible species with the highest average risk of HSE are arctic foxes (average RR = 64.2), raccoon dogs (54.5), dholes (52.2), corsac foxes (*Vulpes corsac*, 33.5), and golden jackals (*Canis aureus*, 29.9). Of these species, only dholes and corsac foxes are not known reservoirs in Asia. The highest risk RVVs are Eurasian Gray Wolf (historic; 2.7), Canine-associated RVVs (2.5), Golden Jackal (1.5), and Raccoon Dog (1.2).

### Europe

In Europe, the highest risk interactions are arctic foxes from Raccoon Dog RVV (RR = 115.8), arctic foxes from Golden Jackal RVV (99.5), corsac foxes from Eurasian Red Fox RVV (45.5), red foxes from Raccoon Dog RVV (45.2), and corsac foxes from Arctic Fox RVV (44.7). There are no documented HSEs from Golden Jackal, Raccoon Dog, or Eurasian Red Fox RVVs.

The susceptible species with the highest average risk of HSE are arctic foxes (average RR = 83.8), corsac foxes (38.0), red foxes (35.2), golden jackals (34.9), and dogs (15.2). Arctic foxes, golden jackals, and red foxes are considered active reservoir species in Europe, and dogs have been reservoirs for historic RVVs. The highest risk RVVs are Eurasian Gray Wolf (historic; 3.6), Canine-associated RVVs (historic; 3.3), Raccoon Dog (2.2), and Golden Jackal (1.9).

### Oceania

Only one species in Oceania is at high risk of an HSE from Canine-associated RVVs: cats (*Felis catus*, RR = 4.4).

## Discussion

This model attempts to characterize the factors that are associated with HSEs based on characteristics of the viral variant, the reservoir species, and the exposed susceptible animal. Several previous studies have explored the risk of rabies HSEs from either a molecular standpoint or limited reservoir species traits, however this is the first model that considers the most influential factors of all three components of a host-shift: the virus, the reservoir, and the susceptible animal^9,10^. The model performs well based on the average accuracy of leave-one-out cross validation, and the average risk among known HSEs is significantly higher than other non-reservoir infections in the model. While the pathway for a successful HSE is complex, and certainly additional ecological factors are important to consider that could not be included in this study, utilizing a multivariable logistic regression model to quantify host-shift risks allows us to explore how translocations and the changing compositions of animal communities globally could change the epidemiology of rabies in ways that increase human rabies risk.

One of the key goals of this model is to predict future HSEs, which can aid rabies programs in determining an adequate level of response when a cross species transmission event of a high-risk reservoir-susceptible infection is detected. For example, the highest risk interaction for an HSE was found when coyotes are infected with Canine-associated RVVs, suggesting that when these events are detected a more robust investigation and contingency action may be necessary. This may include peri-focal vaccination programs, community education about risks and reporting suspected cases, or even in extreme instances localized de-population of the susceptible species^22,23^. Other high-risk infections that should raise alarms when identified include gray wolf and African wild dogs that are infected with the Canine-associated RVVs, corsac foxes infected with Eurasian Red Fox RVV, and foxes (red and Cape [*Vulpes chama*]) infected with either of the African Jackal RVVs (Table 3).

When considering the HSE-risk by variant, the Canine-associated RVVs have the highest cumulative risk of causing an HSE. This should raise concern for countries where Canine-associated RVVs are endemic, as this suggests that the longer dog-mediated rabies persists, the more chances there are for an HSE to occur and for RABV cycles to become established in wildlife populations. As has been experienced in the Western Hemisphere, sylvatic transmission of rabies virus is challenging and costly to control, leading to long-term needs for vaccination programs in humans and domestic animals^24^. However, this model highlights that dogs are not the only species of concern regarding the prevention of future HSEs. In the Western Hemisphere, there was relatively high cumulative risk of an HSE from the Coyote, Raccoon, Skunk, and Fox RVVs. In Africa, Asia, and Europe, Jackal RVVs represent the greatest non-Canine-associated RVVs risk for causing the next HSE. Every continent except for Australia and Antarctica has at least one rabies reservoir species that was found to have an increased risk for causing a future HSE; rabies control programs should consider these risks and establish contingency plans if these susceptible-reservoir infections are detected.

High-risk species for maintaining cryptic rabies cycles, those species that may maintain RABV cycles that have gone undetected by rabies programs, can also be identified from this model. Areas lacking robust wildlife surveillance are likely to miss detection of wildlife-maintained RVVs, while rabies programs with robust surveillance systems and variant typing capabilities are less likely to have cryptic reservoirs present, since they have adequate resources to detect when an HSE may be occurring. Minhaj et al. found that wildlife rabies testing rates were 10–100-fold lower in countries enzootic for the Canine-associated RVVs, compared to those countries that have controlled dog-mediated rabies^25^. Our model results should raise concern for the likelihood of undiscovered cryptic wildlife rabies reservoirs, particularly in Central America, central South America, Sub-Saharan Africa, and much of Asia, which have higher cumulative risks for HSEs and have low rates of wildlife surveillance^25^. As rabies programs in these areas begin to increase wildlife surveillance efforts, results from this model can be used to identify high-risk susceptible species for focused surveillance efforts, thereby increasing probability for detection while decreasing surveillance costs.

This model may also inform the implications of imported RVVs. In areas of the Americas and Europe where dog-mediated rabies has been eliminated, low levels of free-roaming dog populations, relatively high vaccination rates, strict dog importation requirements, and other precautions help reduce the risk of Canine-associated RVVs becoming re-established in dog populations. However, this model indicates that several wildlife species in North America and Europe could be at high risk if an imported rabid dog were to be introduced into the ecosystem. Coyotes, gray wolves, skunks, and several species of foxes infected by Canine-associated RVVs are all among the highest risk for an HSE in North America and Europe according to the model (Table 3). HSEs from dogs have already occurred in several of these species resulting in the active Artic Fox, Arizona/Mexico Gray Fox, North Central Skunk, California Skunk RVVs and the now-extinct Texas/Mexico Coyote and North American Red Fox RVVs^2,26–28^.

Another concern this model can help identify is the expansion of existing RVV territories, which might place new species at risk for HSEs. Raccoons are responsible for many predicted high-risk interactions in North America. The Eastern Raccoon RVV is maintained by raccoons in the eastern USA, but raccoons inhabit a majority of North and Central America where this RVV is not present. Vaccination and enhanced surveillance efforts along the RVV territory boundary help to prevent the westward spread of this variant^29^. The results of this model suggest that if the Eastern Raccoon RVV were to expand westward, species like hooded skunks, ringtails (*Bassariscus astutus*), hog-nosed skunks (*Conepatus leuconotus*), spotted skunks (*Spilogale putorius*), crab-eating raccoons (*Procyon cancrivorus*), swift foxes, kit foxes (*Vulpes macrotis*), and coyotes, would be highly susceptible to an HSE. Several studies have considered the cost-benefit of Eastern Raccoon RVV control programs based on the increased costs of vaccines (human and animal) and loss-of-life (human and animal) if the variant were to become endemic throughout North America, but these studies have not considered the downstream impacts of an HSE into a new wildlife species^29^. The results from this model suggest that an additional benefit of this control program, and other programs like it (e.g., red fox rabies control programs in Europe), is to prevent a catalyst of HSEs in these high-risk susceptible species.

The shifting of species’ habitat ranges due to climate change may create conditions for future HSEs. These model results could be useful to identify high-risk infections if habitat or RVV territory changes. The current model considers interactions between all mammalian species found on the continent, but in many instances these reservoir-susceptible species do not have overlapping ecosystems. Therefore, the model could be useful to understand the potential risks should environmental changes lead to these habitat overlaps. Current climate models suggest that warming temperatures may result in increases in the extent of red foxes in the polar Arctic, increasing the risk for circumpolar expansion of the Arctic Fox RVV through a red fox HSE^30,31^. The model suggests that circumpolar Arctic warming could increase the risk of an HSE into red foxes, coyotes, and gray wolves in North America, Europe, and Asia. Similarly, the habitat range of hematophagous bats (*Desmodus rotundus*) may be expanding north into the United States^32^. Striped skunks, red foxes, and gray foxes are all at high risk of an HSE from *Desmodus rotundus* RVV. In these examples, the involved species currently have limited opportunities for interaction and virus-sharing but may begin to come into contact more frequently as climate change alters ecosystems, potentially leading to the establishment of new RVVs.

Rabies outbreaks have been shown to put endangered species at risk of extinction, particularly endangered canid populations like the Ethiopian wolf (*Canis simensis*) and African wild dogs^33^. As previously mentioned, this model does not consider certain ecological factors such as low population density which would likely prohibit an HSE from becoming established, as would likely be expected if RABV were introduced into an endangered animal population. However, this model can be used to identify endangered species that are at high risk of significant losses or even extinction if RABV were introduced into the population. Notably, fossa (*Cryptoprocta ferox*) in Madagascar, dhole in Asia, and African wild dogs in southern Africa are considered Vulnerable, Endangered, and Critically Endangered on the IUCN Red List and are among the highest-risk species for an HSE^1^. Vaccinating reservoir species is often conducted to protect endangered species, and this model could provide additional supporting evidence to continue these vaccination practices where high-risk enzootic variants are circulating.

Because this model aims to quantify risk based on the biological relationships between susceptible and reservoir species, environmental factors, such as urbanization and landscape features, are not incorporated. However, the results of the model can be used to help us better understand the potential impact these factors have on rabies HSEs. Land use change affects not only how humans and wildlife interact, but also how wildlife interacts with each other. This has been seen in Arizona, USA where skunks have become an intermittent RABV reservoir species resulting from interactions with *Eptesicus fuscus* bats^3^. Infrastructure and amenity development in Flagstaff, Arizona affect interactions between terrestrial wildlife and bats in the area^34^. According to the model, skunks infected by *Eptesicus fuscus* RVVs are at high risk for an HSE. This real-world example not only provides supporting evidence for the validity of this HSE model, but it now also provides a quantitative measure which can inform future contingency actions.

The results of this model can be used in a variety of ways depending on the rabies status and surveillance capacity of the country or programmatic area. For rabies programs that have eliminated dog-mediated rabies and have established robust surveillance systems, this model can be considered when a high-risk infection is identified to assist in the decision of appropriate response actions. The term “contingency action” can involve numerous response activities including “wait and watch”, increased surveillance efforts, focused vaccination efforts, or population reduction (although this is typically only considered appropriate for wildlife management purposes). While the appropriate level of a contingency action is often based on expert consensus and resource availability, this model now provides a quantitative measure which adds additional information to these decisions; an infection with a low RR for HSE may influence programs to adopt more of a “wait and see” approach, whereas a high RR infection may lead to more active and costly measures, implemented earlier.

The intention of this model was to characterize HSE risks globally, therefore we were limited to only considering variables that have data available for the majority of mammalian species; this limited the types of factors that could be considered, particularly ecological factors. The population density of susceptible species is an important factor in whether RABV will be able to be maintained in that population and is not captured in this model^33,34^. A lack of widely available data prevented the inclusion of population density as a model parameter.

Due to a lack of wildlife surveillance in many parts of the world, this model likely does not contain every active RVV and certainly does not have every abortive HSE. North America’s robust wildlife surveillance system has discovered numerous RVVs maintained through wildlife reservoirs, and even with advanced surveillance capacities, new cryptic transmission cycles are still being discovered^35^. It is likely that there are cryptic RVV transmission cycles in wildlife throughout other parts of the world that are waiting to be uncovered. While this model can be useful for identifying species that may be maintaining these cryptic cycles, this is also a limitation when analyzing the results of this model. Since there are fewer known RVVs in Africa and Asia, the model includes a lower number of potential susceptible-reservoir infections and therefore is likely under-estimating the cumulative HSE risk on these continents. As surveillance for wildlife improves and new reservoirs are detected, this model can be updated to reflect the changing risks.

Host-shift events have been occurring for thousands of years and have led to the current diverse landscape of rabies virus variants and reservoir species. Reservoir-susceptible animal interactions will continue to occur, and changing climates and ecosystems will only increase the diversity of these interactions. Understanding not only the risk that these interactions have for an HSE, but also the geographic locations and species at highest risk to be involved in such an event will shape how future surveillance programs operate. This is the first HSE risk model to consider all three critical components of the HSE pathway and to apply a model like this to the majority of mammalian species and rabies virus variants found globally. As such, these findings reinforce the urgency to eliminate Canine-associated RVVs before they shift into other species, inform more targeted strategies to uncover cryptic rabies cycles, and provide cautionary evidence about the potential impact of climate change and the introduction of rabies viruses into endangered species populations.

## Electronic supplementary material

Below is the link to the electronic supplementary material.

Supplementary Material 1

Supplementary Material 2

## Data Availability

All data was gathered from previously published sources and is available in the supplementary Dataset S1.
