# Supplementary material for: A logistic regression model to predict the next rabies virus host-shift event
Source: Sci Rep. 2025 Jun 2;15:19306. doi: 10.1038/s41598-025-98986-x (PMC12130182; doi:10.1038/s41598-025-98986-x)
Supplement: Supplementary file 2 — Supplementary Material 2 [file 41598_2025_98986_MOESM2_ESM.docx]

**Supplementary information**

**A logistic regression model to predict the next rabies virus host-shift event**

# Cassandra Boutelle^1^*, Nardus Mollentze^2, 3^, Crystal Gigante^1^, Felipe Rocha^4^, Marco A. N. Vigilato^4^, Daniel G. Streicker^2, 3^, Ryan Wallace^1^

^1^ United States Centers for Disease Control and Prevention, Poxvirus and Rabies Branch, Atlanta, GA, United States

^2^ School of Biodiversity, One Health and Veterinary Medicine, University of Glasgow, Glasgow G12 8QQ, United Kingdom

^3^ MRC-University of Glasgow Centre for Virus Research, Glasgow G61 1QH, United Kingdom

^4^ Pan American Center for Foot and Mouth Disease and Veterinary Public Health-PANAFTOSA/VPH-PAHO/WHO, Rio de Janeiro, Brazil.

*qou7@cdc.gov

| **Terrestrial Rabies virus reservoir species and variants associated with host shift events** | | | | | | |
| --- | --- | --- | --- | --- | --- | --- |
| **Region** | **Reservoir species** | **Model Variant Name** | **Rabies Virus Variant(s)** | **Lineage** | **Status**** | **Evidence** |
| North & Central America and Caribbean | Raccoon (*Procyon lotor*) | Eastern raccoon | Eastern Raccoon | Bat | Active | Phylogenetic and Epidemiologic [1] |
|  | Striped skunk (*Mephitis mephitis*) | North Central skunk | Cosmopolitan (AM1) | Canine | Active | Phylogenetic and Epidemiologic [1] |
|  | Striped skunk (*Mephitis mephitis*) | South Central skunk | USA South Central Skunk | Bat | Active | Phylogenetic and Epidemiologic [1] |
|  | Striped skunk (*Mephitis mephitis*) | California skunk | Cosmopolitan (AM4) | Canine | Active | Phylogenetic and Epidemiologic [1] |
|  | Striped skunk (*Mephitis mephitis*) | Flagstaff skunk* | *Eptesicus fucscus* W1 | Bat | Intermittent | Phylogenetic and Epidemiologic [2] |
|  | Spotted skunk (*Spilogale putorius*) | Baja California skunk | Baja California Skunk (V10) | Canine | Active | Phylogenetic and Epidemiologic [3, 4] |
|  | Spotted skunk (*Spilogale putorius*) | Central Mexico skunk | Central Mexico skunk (V8) | Bat | Active | Phylogenetic and Epidemiologic [3, 4] |
|  | Spotted skunk (*Spilogale putorius*) | Sonora skunk | Sinaloa-Durango Skunk (V1) | Canine | Active | Phylogenetic and Epidemiologic [5, 6] |
|  | Mongoose (*Urva auropunctata*) | Puerto Rico/Dominican Republic mongoose | Cosmopolitan (AM1-like) | Canine | Active | Phylogenetic and Epidemiologic [1, 7] |
|  | Mongoose (*Urva auropunctata*) | Grenada mongoose | Cosmopolitan (Europe/Middle Eastern-like) | Canine | Active | Phylogenetic and Epidemiologic [7, 8] |
|  | Mongoose (*Urva auropunctata*) | Cuba mongoose | Cosmopolitan (AM2-like) | Canine | Active | Phylogenetic and Epidemiologic [7, 8] |
|  | Gray fox (*Urocyon cinereoargenteus*) | Arizona/Mexico gray fox (V7) | Cosmopolitan (AM2b) | Canine | Active | Phylogenetic and Epidemiologic [1, 4] |
|  | Gray fox (*Urocyon cinereoargenteus*) | Texas gray fox | Cosmopolitan (AM2) | Canine | Anthropogenic extinction | Phylogenetic and Epidemiologic [9] |
|  | Gray fox (*Urocyon cinereoargenteus*) | California gray fox | Cosmopolitan (AM4) | Canine | Abortive | Phylogenetic and Epidemiologic [10] |
|  | Gray fox (*Urocyon cinereoargenteus*) | Oregon gray fox* | *Eptesicus fucscus* W2 | Bat | Abortive | Epidemiologic [11] |
|  | Gray fox (*Urocyon cinereoargenteus*) | Maine gray fox* | Eastern Raccoon | Bat | Abortive | Epidemiologic [12] |
|  | Arctic Fox (*Vulpes lagopus*) | Arctic fox | Arctic-related | Canine | Active | Phylogenetic and Epidemiologic [2] |
|  | Red fox (*Vulpes vulpes*) | North American red fox | Cosmopolitan | Canine | Natural extinction | Phylogenetic and Epidemiologic [1] |
|  | Red fox (*Vulpes vulpes*) | Ontario red fox | Arctic-related | Canine | Anthropogenic extinction | Epidemiologic [13] |
|  | Coati (*Nasua narica*) | Yucatan coati* | *Tadarida brasiliensis* | Bat | Active | Epidemiologic [14] |
|  | Coyote (*Canis latrans*) | Texas/Mexico Coyote | Cosmopolitan (AM2a) | Canine | Anthropogenic extinction | Phylogenetic and Epidemiologic [15] |
|  | Dog (*Canis lupus familiaris*) | Canine-associated variants | Cosmopolitan (AM2a, AM1-like) | Canine | Active | Phylogenetic and Epidemiologic [9] |
| South America | Crab-eating fox (*Cerdocyon thous*) | Brazil crab-eating fox | Cosmopolitan (AM3b) | Canine | Active | Phylogenetic and Epidemiologic [16] |
|  | Hoary fox (*Lycalopex vetulus*) | Brazil hoary fox | Cosmopolitan (AM3b) | Canine | Active | Phylogenetic and Epidemiologic [17] |
|  | Dog (*Canis lupus familiaris*) | Canine-associated variants | Cosmopolitan (AM3a) | Canine | Active | Phylogenetic and Epidemiologic [16] |
| Africa | Yellow mongoose (*Cynictis penicillata*) | Africa mongoose | Africa-3 | Canine | Active | Epidemiologic [18] |
|  | Bat-eared fox (*Otocyon megalotis*) | Bat-eared fox | Cosmopolitan (AF1b) | Canine | Active | Phylogenetic and Epidemiologic [19] |
|  | Black-backed jackal (*Canis mesomelas*) | Black-backed jackal | Cosmopolitan (AF1a) | Canine | Active | Phylogenetic and Epidemiologic [20, 21] |
|  | Side-striped jackal (*Canis adustus*) | Side-striped jackal | Cosmopolitan | Canine | Active | Epidemiologic [20] |
|  | Dog (*Canis lupus familiaris*) | Canine-associated variants | Cosmopolitan (AF1, AF4), Africa-2 | Canine | Active | Phylogenetic and Epidemiologic [16] |
| Eurasia | Indian gray mongoose (*Herpestes edwardsi*) | India mongoose | Unknown | Canine | Active | Epidemiologic [22] |
|  | Ferret-badger (*Melogale moschata*) | Ferret-badger | SEA2b, SEA5 | Canine | Active | Phylogenetic and Epidemiologic [23] |
|  | Golden jackal (*Canis aureus*) | Golden jackal | Cosmopolitan (ME1) | Canine | Active | Epidemiologic [24] |
|  | Raccoon dog (*Nyctereutes procyonoides*) | Raccoon dog-associated variants | Cosmopolitan (NEE), Arctic-related | Canine | Active | Epidemiologic [25] |
|  | Arctic Fox (*Vulpes lagopus*) | Arctic fox | Arctic-related | Canine | Active | Phylogenetic and Epidemiologic [16] |
|  | Red fox (*Vulpes vulpes*) | Eurasian red fox | Cosmopolitan (CE, EE, NEE, WE, ME1) | Canine | Near anthropogenic extinction | Epidemiologic [25] |
|  | Red fox (*Vulpes vulpes*) | Turkey red fox | Cosmopolitan (ME2) | Canine | Abortive | Phylogenetic and Epidemiologic [26] |
|  | Gray wolf (*Canis lupus*) | Eurasian gray wolf | Unknown | Canine | Natural extinction | Epidemiologic [27] |
|  | Dog (*Canis lupus familiaris*) | Canine-associated variants | Cosmopolitan (CA1, CA2, CA3, AF4, ME1, ME2), Arctic-related, Indian-subcontinent, SEA1, SEA2a, SEA3, SEA4 | Canine | Active | Phylogenetic and Epidemiologic [16] |
| Additional species such as marsh mongoose, spotted hyena, golden palm civet, meerkat, Peruvian fox, kinkajou, marmoset, and corsac fox have been considered RABV reservoirs by other studies but were excluded from this list due to lack of sufficient phylogenetic and/or epidemiologic evidence. This is not a complete list of RVVs; Rabies virus variants circulated by reservoir species conform to naming in Troupin et al, Kuzmin et al, and Streiker et al, where possible [28-30].  * Rabies virus variant did not diverge significantly from the “mother” variant to form a new variant  ** Active status indicates current maintenance by the reservoir species. Natural extinction indicates that RABV was once maintained by the reservoir species, but that RVV naturally died out without human intervention. Anthropogenic extinction indicates that RABV was once maintained by the reservoir species, but that RVV was eliminated by humans through vaccination or other means. Abortive indicates that there is epidemiologic or phylogenetic evidence of a new RVV in the reservoir species, but the RVV was not maintained by the species for a prolonged period of time. Intermittent indicates that there is epidemiologic or phylogenetic evidence of a new RVV in the reservoir species, and the RVV is not consistently observed, but re-emerges in cycles. | | | | | | |

**Table S1.** Recognized terrestrial RVVs and their respective reservoir species.

| **Common bat Rabies virus reservoir species** | | |
| --- | --- | --- |
| **Reservoir species** | **Region** | |
|  | **North/Central America** | **South America** |
| *Antrozous pallidus* | ✓ |  |
| *Artibus lituratus* | ✓ | ✓ |
| *Corynorhinus townsendii* | ✓ |  |
| *Desmodus rotundus* | ✓ | ✓ |
| *Eptesicus brasiliensis* |  | ✓ |
| *Eptesicus furinalis* | ✓ | ✓ |
| *Eptesicus fuscus* | ✓ | ✓ |
| *Lasiurus borealis* | ✓ |  |
| *Lasiurus cinereus* | ✓ | ✓ |
| *Lasiurus intermedius* | ✓ |  |
| *Lasiurus noctivagans* | ✓ |  |
| *Lasiurus seminolus* | ✓ |  |
| *Lasiurus xanthinus* | ✓ |  |
| *Macrophyllum macrophyllum* | ✓ |  |
| *Molossus molossus* | ✓ | ✓ |
| *Myotis californicus* | ✓ |  |
| *Myotis evotis* | ✓ |  |
| *Myotis keenii* | ✓ |  |
| *Myotis leibii* | ✓ |  |
| *Myotis lucifugus* | ✓ |  |
| *Myotis septentrionalis* | ✓ |  |
| *Myotis sp.* | ✓ |  |
| *Myotis thysanodes* | ✓ |  |
| *Myotis volans* | ✓ |  |
| *Myotis yumanensis* | ✓ |  |
| *Mytois ciliolabrum* | ✓ |  |
| *Nycticeius humeralis* | ✓ |  |
| *Nyctinomops ssp.* | ✓ | ✓ |
| *Parastrellus hesperus* | ✓ |  |
| *Pipistrellus subflavus* | ✓ | ✓ |
| *Tadarida brasiliensis* | ✓ |  |
| *Histiotus sp.* |  | ✓ |

**Table S2.** List of known bat reservoir species in the United States and their respective RVVs. Reservoir presence in South America is assumed if the habitat of that species extends to South America [28, 31].

| **Parameter** | | **Estimate** | **2.5%** | **97.5%** |
| --- | --- | --- | --- | --- |
| Intercept | | 116.5 | -29.3 | 262.2 |
| Relatedness | | 0.0 | 0.0 | 0.0 |
| Temperature | | -0.2 | -0.4 | 0.1 |
| Temperature difference | | -0.4 | -0.9 | 0.1 |
| Litter size | | 1.9 | 1.0 | 2.9 |
| Weight | | 0.8 | 0.4 | 1.2 |
| Weight difference | Low | -3.9 | -6.4 | -1.4 |
|  | High | 1.4 | 0.4 | 2.4 |
| Lineage | | 0.7 | -0.4 | 1.9 |

**Table S3.** Final model parameter estimates with 95% confidence intervals. Parameters with confidence intervals that cross 0 were still deemed to be critical to evaluating the risk of an HSE.

# References

1. Ma, X., et al., *Rabies surveillance in the United States during 2021.* J Am Vet Med Assoc, 2023. **261**(7): p. 1045-1053.

2. Kuzmin, I.V., et al., *Molecular inferences suggest multiple host shifts of rabies viruses from bats to mesocarnivores in Arizona during 2001-2009.* PLoS Pathog, 2012. **8**(6): p. e1002786.

3. Davis, R., et al., *Genetic characterization and phylogenetic analysis of skunk-associated rabies viruses in North America with special emphasis on the central plains.* Virus Res, 2013. **174**(1-2): p. 27-36.

4. Velasco-Villa, A., et al., *Antigenic diversity and distribution of rabies virus in Mexico.* J Clin Microbiol, 2002. **40**(3): p. 951-8.

5. Velasco-Villa, A., et al., *Molecular epizootiology of rabies associated with terrestrial carnivores in Mexico.* Virus Res, 2005. **111**(1): p. 13-27.

6. Garces-Ayala, F., et al., *Rabies Virus Variants Detected from Cougar (Puma concolor) in Mexico 2000-2021.* Pathogens, 2022. **11**(2).

7. Sauve, C.C., et al., *Home range overlap between small Indian mongooses and free roaming domestic dogs in Puerto Rico: implications for rabies management.* Sci Rep, 2023. **13**(1): p. 22944.

8. Zieger, U., et al., *The phylogeography of rabies in Grenada, West Indies, and implications for control.* PLoS Negl Trop Dis, 2014. **8**(10): p. e3251.

9. Rohde, R.E., et al., *Molecular epidemiology of rabies epizootics in Texas.* Clin Diagn Virol, 1997. **8**(3): p. 209-17.

10. Borucki, M.K., et al., *Ultra-deep sequencing of intra-host rabies virus populations during cross-species transmission.* PLoS Negl Trop Dis, 2013. **7**(11): p. e2555.

11. Blanton, J.D., et al., *Rabies surveillance in the United States during 2010.* J Am Vet Med Assoc, 2011. **239**(6): p. 773-83.

12. Wildlife, M.D.o.I.F.a., *MDIFW NEWS: MDIFW, USDA Wildlife Services, City of Bath Working Together On Rabies Issue In Bath*. 2020: Maine Department of Inland Fisheries and Wildlife.

13. Andrew, B.C., *The Ecology of Red Foxes, Gray Foxes, and Rabies in the Eastern United States , urldate = 2023-09-28.* Wildlife Society Bulletin (1973-2006), 1982. **10**(1): p. 18--26.

14. Rosatte, R.C., et al., *Elimination of arctic variant rabies in red foxes, metropolitan Toronto.* Emerg Infect Dis, 2007. **13**(1): p. 25-7.

15. Arechiga-Ceballos, N., et al., *New rabies virus variant found during an epizootic in white-nosed coatis from the Yucatan Peninsula.* Epidemiol Infect, 2010. **138**(11): p. 1586-9.

16. Wallace, R.M. and J. Blanton, *Epidemiology*, in *Rabies*. 2020. p. 103-142.

17. Caraballo, D.A., et al., *A Novel Terrestrial Rabies Virus Lineage Occurring in South America: Origin, Diversification, and Evidence of Contact between Wild and Domestic Cycles.* Viruses, 2021. **13**(12).

18. Koeppel, K.N., O.L. van Schalkwyk, and P.N. Thompson, *Patterns of rabies cases in South Africa between 1993 and 2019, including the role of wildlife.* Transbound Emerg Dis, 2022. **69**(2): p. 836-848.

19. Sabeta, C.T., et al., *Molecular epidemiology of rabies in bat-eared foxes (Otocyon megalotis) in South Africa.* Virus Res, 2007. **129**(1): p. 1-10.

20. Bingham, J., et al., *The epidemiology of rabies in Zimbabwe. 2. Rabies in jackals (Canis adustus and Canis mesomelas).* Onderstepoort J Vet Res, 1999. **66**(1): p. 11-23.

21. Zulu, G.C., C.T. Sabeta, and L.H. Nel, *Molecular epidemiology of rabies: focus on domestic dogs (Canis familiaris) and black-backed jackals (Canis mesomelas) from northern South Africa.* Virus Res, 2009. **140**(1-2): p. 71-8.

22. Mani, R.S., et al., *Rabies following mongoose bite.* Indian J Med Microbiol, 2016. **34**(2): p. 256-7.

23. Chiou, H.Y., et al., *Molecular characterization of cryptically circulating rabies virus from ferret badgers, Taiwan.* Emerg Infect Dis, 2014. **20**(5): p. 790-8.

24. Yakobson, B.A., et al., *Assessment of the efficacy of oral vaccination of livestock guardian dogs in the framework of oral rabies vaccination of wild canids in Israel.* Dev Biol (Basel), 2008. **131**: p. 151-6.

25. Vos, A., et al., *Oral vaccination of foxes and raccoon dogs against rabies with the 3rd generation oral rabies virus vaccine, SPBN GASGAS, in Finland.* Acta Vet Scand, 2021. **63**(1): p. 40.

26. Marston, D.A., et al., *Genetic analysis of a rabies virus host shift event reveals within-host viral dynamics in a new host.* Virus Evol, 2017. **3**(2): p. vex038.

27. Baltazard, M. and M. Ghodssi, *Prevention of human rabies; treatment of persons bitten by rabid wolves in Iran.* Bull World Health Organ, 1954. **10**(5): p. 797-803.

28. Streicker, D.G., et al., *Host phylogeny constrains cross-species emergence and establishment of rabies virus in bats.* Science, 2010. **329**(5992): p. 676-9.

29. Kuzmin, I.V., et al., *Bats, emerging infectious diseases, and the rabies paradigm revisited.* Emerg Health Threats J, 2011. **4**: p. 7159.

30. Troupin, C., et al., *Large-Scale Phylogenomic Analysis Reveals the Complex Evolutionary History of Rabies Virus in Multiple Carnivore Hosts.* PLoS Pathog, 2016. **12**(12): p. e1006041.

31. Campbell, K., et al., *Making genomic surveillance deliver: A lineage classification and nomenclature system to inform rabies elimination.* PLoS Pathog, 2022. **18**(5): p. e1010023.
